# Supplementary material for: The Influence of Organizational Aspects of the U.S. Agricultural Industry and Socioeconomic and Political Conditions on Farmworkers’ COVID-19 Workplace Safety
Source: Int J Environ Res Public Health. 2023 Dec 3;20(23):7138. doi: 10.3390/ijerph20237138 (PMC10706775; doi:10.3390/ijerph20237138)
Supplement: Supplementary file 1 [file ijerph-20-07138-s001.zip › ijerph-2685845-supplementary.pdf]

ENGLISH LANGUAGE VERSION

[OPENING: OBTAINING A GENERAL SENSE OF HOW COVID IS CURRENTLY AFFECTING THEM]

First, I would like to learn a little bit about you.

- Tell me little about yourself and your family?

[WORK AND EMPLOYMENT]

We are interested in learning about the changes you have experienced in your employment or at work during the pandemic.

- Tell me about your job?
- What kind of word do your parents do?
    - [If appropriate] How did you find this job?
    - What do you like about your work?
    - What don't you like about your job?
    - How do you get along with your coworkers? Your bosses?
    - Are you in a union?
        - [If yes] Tell me about your union.
- What are your responsibilities at work?
- Have you lost a job or been unemployed this year?
    - [If yes] What did you do during that time?
- What concerns do you have about your health when you are at work?
    - Have you ever communicated your concerns to your employer? What happened?
    - How do you stay safe from COVID-19 at work?
        - What personal protective equipment has your employer provided you at work? Where do you get it?
        - What protections have been implemented in your work during the pandemic?
        - How do your coworkers protect themselves at work?
- In the last year, have you missed work for any reason?
    - Why? What happened?
- Have there been any cases of coronavirus in your work?
    - What did your company do?
    - How did you feel? What did you do?

[HOUSEHOLD]

We'd like to know how your expenses have been affected by the pandemic.

- What are your biggest expenses right now?
    - What difficulties have you faced in covering these expenses?
        - [Specifically] What assistance programs has this year used to maintain household expenses?
    - Who do you trust for financial support?
    - What do you do when you can't find or get financial support?
- How has your household income changed this year?
    - For example, have the people in your household lost their jobs?

    - What do you do when you lose income?
    - How do you recover lost income?
- What kind of financial decisions have you had to make during the pandemic?
    - [If clarification is needed] For example, have you had to cut home services to save money?

[CHILDREN & EDUCATION]
We want to learn about your experience with online learning/ you children going back to school.

- How are your kids/you doing at school? How do they feel?
    - What is the most difficult part about remote learning?
    - What communication have you received from the school about online learning?
    - How have you adjusted to online classes?
    - What are you studying? What classes are you taking?
    - How have professors treated you? Can you tell me about a recent interaction you've had with your profesor?
    - What do your children do when you're not in class/with your free time?
    - How do you stay active during this time?
- How do you connect to your classes online?
    - What are the biggest challenges you've encountered during online learning?
        - **[INTERNET]**
            - What difficulties have you encountered with the internet?
            - What do you do when you have problems with the internet?
            - What Internet resources have you used?
- What kind of support have you found for online learning?
    - From your school? Teachers? [technology, internet, etc.]
- How do your children/you feel about online learning?
- What has been your experience with school meals?
- [If the kids are attending school in person]
    - How do you feel about your children returning to school?
    - What is the most difficult part about your children going back to school?
    - What concerns do you have about your health or your children's health?
    - What do you think of the school's decision to reopen schools?
    - How do you prepare your children for school during the pandemic?

[FOOD]
Food is very important for your health. We'd like to know how your groceries or meals have changed.

- What are the meals you eat at home?
    - What changes have you had to make to the meals you make? Why?
        - How does your family feel about these changes?
        - What kind of foods are you buying the most? Less?
        - How have these changes affected you?
            - Have you noticed any changes in your weight, or the weight of your family members?
    - Have you used more canned foods, dry foods, or canned foods?
- How have your food expenses changed this year?

- Can you give me examples of food assistance programs you've been able to use this year?
    - [If they ask for clarification or don't mention] For example, food stamps, or food donations.
    - [If none] What do you do to support your food expenses?
    - How has your experience been with food donations?
- How have you had difficulty buying food for your family during this time? Examples?

[COMMUNITY]
We are interested to know how the pandemic has affected your community.

- What changes have you noticed in your community during the pandemic?
- How do you connect with your friends and community during the pandemic?
    - How do you feel about this?
- How do you help each other among your community? Example?
- What kind of support do you trust your community for?
- What is an example of a time when you sought help or support from your community or friend during the pandemic?
    - How did you feel?

[HEALTH]
We want to know how the pandemic has impacted your health and your family's health.

- What changes have you noticed in your mental health or your family's mental health?
        - [If clarification is needed] Have you noticed, for example, that you feel more anxious?
        - What are the behavioral changes you've noticed in your children?
    - Can you give me an example of when you or someone in your family felt anxious or depressed during this past year?
        - What do I do in that situation?
        - What health resources or support do you use when you feel that way?
- How do you and your family protect yourselves during the pandemic?
    - Where do you get personal protection? For example, masks.
- What concerns do you have about your health? Your family's health?
- Do you have a chronic condition?
    - [If yes] Can you tell me about this?
    - What concernd do you have about your chronic condition during the pandemic?
- Describe a time when you have had an appointment with a clinic since the pandemic began.
    - How'd it go?
- Have you had an appointment online or over the phone?
    - How'd it go?
- People are dealing differently with this moment in time. How are you coping?
    - [Clarification] What do you do to lift the mood?
    - What has helped you manage your problems with ____ during the pandemic?

[CLOSING]
Finally, there is a lot of news about several COVID vaccines that may soon be available to Tulare/Imperial/Fresno/residents.

- What have you heard about the vaccine? How do you feel about it?
- Will you receive the vaccine if it becomes available?
- Why? Why not?

Thank you for sharing your experiences. Is there anything else you'd like to add about your experience?

SPANISH LANGUAGE VERSION

[OPENING: OBTAINING A GENERAL SENSE OF HOW COVID IS CURRENTLY AFFECTING THEM]

Primero me gustaría escuchar sobre sus experiencias durante los últimos meses.

- Ha pasado casi un año desde que se implemento la orden de permanecer en casa. ¿Como se siente?
- ¿Donde has estado viviendo desde que comenzó la pandemia?
- ¿Platíqueme poquito de usted y su familia?
  - ¿A que se dedica usted? ¿Su pareja?
  - ¿En que grado van sus hijos?
  - ¿Como te sientes llendo a la escuela en linea?

[WORK AND EMPLOYMENT]

Estamos interesados en conocer los cambios que ha sentido en su trabajo durante la pandemia.

- ¿Que tipo trabajo hace usted?
  - [If appropriate] Como encontró este trabajo?
  - ¿Que le gusta de su trabajo?
  - ¿Que no le gusta de su trabajo?
  - ¿Como se lleva con sus compañeros? ¿Sus patrones?
  - ¿Esta en una unión? Platíqueme de su unión.
- ¿Cuales son sus deberes o responsabilidades en el trabajo?
  - ¿Con cuantas personas trabaja?
- ¿Ha perdido trabajo o ha estado desempleado este año?
  - [If yes] ¿Que hico durante ese tiempo?
- ¿Qué preocupaciones tiene sobre su salud cuando está en el trabajo?
  - ¿Qué equipo de protección personal le ha brindado su empleador en el trabajo?
  - ¿Que protecciones se han implementado en su trabajo durante la pandemia?
  - ¿Como se protege usted mismo del coronavirus en el trabajo?
  - ¿Como se protegen sus compañeros en el trabajo?
- ¿En el ultimo año ha faltado ir ha trabajar por cualquier razón?
  - ¿Por que? ¿Que fue lo que paso?
- ¿Ha habido casos de coronavirus en su trabajo?
  - ¿Que izo en esa situación?
  - Que izo su patrón/empresa en esa situación?

[HOUSEHOLD EXPENSES]

Nos gustaría saber como ha afectado al pandémica sus gastos.

- ¿Cuales son sus gastos mas grandes en este momento?
- ¿Que dificultades ha enfrentado para cubrir estos gastos?
  - [Specifically] ¿Cuales programas de asistencia ha usado este ano para mantener los gastos del hogar?
  - ¿En quién confía para obtener apoyo financiero?
  - ¿Qué hace cuando no puede encontrar u obtener apoyo financiero?
- ¿Cómo han cambiado los ingresos de su hogar este año?

- ¿Han perdido su trabajo las personas de su hogar?
    - ¿Que es lo que hace cuando pierde ingresos?
    - ¿Como recupera ingresos perdidos?
- ¿Cuales decisiones financieras ha tenido que tomar durante la pandemia?
    - [If clarification is needed] Por ejemplo, ¿Ha tenido que cortar servicios del hogar para ahorrar dinero?

[CHILDREN & EDUCATION]

Queremos conocer su experiencia con el aprendizaje en línea o con el regreso a las clases.

- ¿Como van sus hijos en la escuela?
    - ¿Como se han ajustado a las clases en línea?
    - ¿Que hacen cuando no están en clase/con su tiempo libre?
    - ¿Como se mantienen activos durante este tiempo?
    - ¿Que estas estudiando? Que clases estas tomando?
    - ¿Cómo te han tratado tus profesores? ¿Puede hablarme de una interacción reciente que ha tenido con tu profesor?
- ¿Como se conectan a sus clases en línea?
    - ¿Cuáles son los mayores desafíos que ha encontrado durante el aprendizaje en línea?
        - **[INTERNET]**
            - ¿Que dificultades ha encontrado con el internet?
            - ¿Que hace cuando tiene problemas con el internet?
            - ¿Cuales recursos de internet a podido usar?
- ¿Qué tipo de apoyo ha encontrado para el aprendizaje en línea?
    - ¿De su escuela? ¿De maestros? [technology, internet, etc.]
- ¿Cómo se sienten sus hijos sobre el aprendizaje en línea?
- ¿Cuál ha sido su experiencia con las comidas escolares?
- [If the kids are attending school in person]
    - ¿Cómo se siente con el regreso de sus hijos a la escuela?
    - ¿Qué preocupaciones tiene sobre su salud o la salud de sus hijos?
    - ¿Qué opina de la decisión de la escuela de reabrir escuelas?
    - ¿Cómo prepara a sus hijos para la escuela durante la pandemia?

[FOOD]

La alimentación es muy importante para la salud. Nos gustaría saber como han cambiado sus comprad de alimentos o comidas.

- ¿Cuales son las comidas que comen en casa?
    - ¿Me puede dar ejemplos de los cambiados que ha sentido en su alimentación?
    - ¿Como han cambiado sus compras de comida?
        - ¿Qué tipo de alimentos está comprando más? ¿Menos?
        - Como se sienten sus hijos/familia con estos cambios?
        - ¿Como les han afectado estos cambios? ¿Han notado cambios en su peso, o en el peso de los miembros de su familia?
    - ¿Ha usado más alimentos enlatados, alimentos secos, o enlatados?
- ¿Cómo han cambiado sus gastos de comida este año?
- ¿Me puede dar ejemplos de los apoyos alimentarios que ha podido usar este año?

    - [If they ask for clarification or don’t mention] Por ejemplo, estampías de comida, o donaciones de comida.
    - [If none] Que hace para apoyar sus gastos alimentarios?
    - ¿Como ha sido su experiencia con las donaciones de comida?
- ¿Cómo se le ha dificultado comprar alimentos para su familia durante este tiempo? ¿Ejemplos?

[COMMUNITY]
Nos interesa saber como ha afectado la pandemia a su comunidad.

- ¿Cuales cambios a notado en su comunidad durante la pandemia?
- ¿Como se conecta con sus amigos y comunidad durante la pandemia?
- ¿Como se ayudan entre su comunidad?
- ¿Para que tipo de apoyo confía en su comunidad?
- ¿Cuál es un ejemplo de un momento en que buscó ayuda o apoyo de su comunidad o amigo durante la pandemia?

[HEALTH]
Queremos saber como la pandemia ha impactado su salud y la salud de su familia.

- ¿Cuáles cambios a notado en su salud mental o en la salud mental de su familia?
        - [If clarification is needed] ¿Por ejemplo, ha notado que se siente mas ansiosa? ¿O que duerme mas o menos?
    - ¿Me puede dar un ejemplo de cuando usted o alguin en su familia se sintió ansioso o deprimido durante este anio pasado?
    - ¿Que sintió?
    - ¿Que hico en esa situación?
    - ¿Cuales recursos de salud o apoyo a podido usar cuando se siente así?
- ¿Como se protege usted y su familia durante la pandemia?
    - ¿En donde consigue protección personal? Por ejemplo, cubre bocas.
- ¿Qué preocupaciones tiene sobre su salud? ¿O la salud de su familia?
- ¿Usted tiene una condición crónica?
    - [If yes] ¿Me puede contar sobre esto?
- Describa un momento en el que tuvo una cita con una clínica desde que comenzó la pandemia.
    - ¿Como le fue?
- ¿Ha tenido una cita en línea o por teléfono? ¿Como le fue?
- La gente está lidiando de manera diferente con este momento en el tiempo. ¿Cómo estas lidiando?
    - [Clarification] Los tiempos son difíciles durante la pandemia. ¿Que hace para levantar el animo?
    - ¿Qué te ha ayudado a manejar tus problemas con ____ durante la pandemia?

[CLOSING]
Finalmente, hay muchas noticias sobre varias vacunas para el COVID que pronto estarán disponibles para los residentes de Tulare/Imperial/Fresno/.

- ¿Que ha oído sobre la vacuna?
- ¿Recibirá la vacuna si se hace disponible?

- ¿Por que? ¿Por que no?

Gracias por compartir sus experiencias. ¿Hay algo mas que le gustaría agregar sobre su experiencia?
